# Benign to Malignant Hepatic Lesion Transformation in Abernethy Malformation

**DOI:** 10.14309/crj.0000000000001307

**Published:** 2024-04-05

**Authors:** Chloe K. Tom, Ramon Ter-Oganesyan, Chopra Shefali, Navpreet Kaur, Jordan L. Pace, Ali Rastegarpour, Yuri Genyk, Jeffrey A. Kahn

**Affiliations:** 1 Division of Gastrointestinal and Liver Diseases, University of Southern California, Los Angeles, CA, USA; 2 Division of Vascular and Interventional Radiology, Department of Radiology, University of Southern California, Los Angeles, CA, USA; 3 Department of Pathology, University of Southern California, Los Angeles, CA, USA; 4 Department of Surgery, University of Southern California, Los Angeles, CA, USA; 5 California University of Science and Medicine Colton, CA, USA; 6 Department of Diagnostic Radiology, University of Southern California Los Angeles, CA, USA; 7 Division of Hepatobiliary/Pancreatic and Abdominal Organ Transplant Surgery, University of Southern California Los Angeles, CA, USA; 8 Division of Gastrointestinal and Liver Diseases and Liver Transplant Program, University of Southern California Los Angeles, CA, USA

**Keywords:** Abernethy malformation, congenital extrahepatic porosystemic shunt, hepatocellular carcinoma, focal nodular hyperplasia, hepatocellular adenoma

## Abstract

Abernethy malformation or congenital extrahepatic portosystemic shunt is an extremely rare condition whereby the portomesenteric blood drains into a systemic vein and bypasses the liver through a complete or partial shunt. Severe complications include hyperammonemia and encephalopathy, benign and malignant liver tumors, and hepatopulmonary syndrome. We describe a case where a female adult diagnosed with congenital extrahepatic portosystemic shunt subsequently developed focal nodular hyperplasia and then hepatocellular carcinoma.

## INTRODUCTION

Abernethy malformation or congenital extrahepatic portosystemic shunt (CEPS) is a rare condition whereby the portomesenteric blood drains into a systemic vein and bypasses the liver through a complete or partial shunt.^1^ The incidence is estimated to be 1:30,000 births and 1:50,000 for those that persist beyond early life.^2^

Complications include hyperammonemia and encephalopathy, benign and malignant liver tumors, hepatopulmonary syndrome, and portopulmonary hypertension.^3^ About 80% of cases are discovered during childhood but may also be diagnosed in adults when complications arise.^4^ We describe a case of an adult female with CEPS and an established history of focal nodular hyperplasia (FNH), who was found to simultaneously have FNH, hepatocellular adenoma (HCA), and HCA, with foci of hepatocellular carcinoma (HCC).

## CASE REPORT

A 12-year-old girl with CEPS presented with premature pubic hair. An ultrasound of the abdomen and pelvis was performed, and hepatic lesions were incidentally discovered. Other than precocious puberty, she did not present with any other symptoms. One lesion was biopsied, demonstrating FNH.

At age 27 years, she presented to the hospital with pruritus. Her vital signs and body mass index were normal. Physical examination was notable for nontender hepatomegaly. Serologies were remarkable for alkaline phosphatase 329 U/L, aspartate aminotransferase 72 U/L, alanine aminotransferase 85 U/L, and ferritin 200 μg/L. Total bilirubin, carcinoembryonic antigen, alpha-fetoprotein (AFP), cancer antigen 19-9, hepatitis C and B, and transthoracic echocardiogram were normal. She had no history of hepatic-related decompensations. She denied any alcohol, tobacco, or drug use, and her family history was unremarkable.

**Figure 1.**
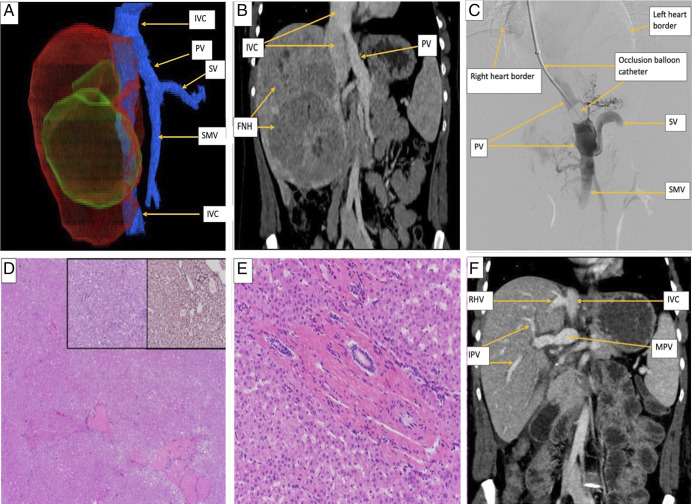
(A) Contrast-enhanced computed tomography with a 3D reconstruction of the abdomen demonstrates the liver and associated venous system in this patient with Type Ib Abernethy malformation. The right lobe of the liver (red) contains multiple masses (green). The associated veins (blue) are comprised of the IVC, SMV, splenic vein, and PV. The SMV and SV join together to form the PV, which in turn drains directly into the extrahepatic IVC, bypassing the liver entirely. (B) Coronal contrast-enhanced computed tomography image of the abdomen demonstrates the portal vein inserting directly into the inferior vena cava. Note the multiple focal nodular hyperplasia masses seen in the right lobe of the liver. (C) Digital subtraction portal venogram performed through a transjugular access balloon catheter. The catheter passes through the following veins/structures to reach the PV (in this order): right jugular vein, right brachiocephalic vein, superior vena cava, right atrium, IVC, and PV. The heart border is seen in the image. The venogram illustrates the direct connection of the PV to the IVC. The inflation of the balloon at the tip of the catheter allows for retrograde contrast opacification of the veins against the direction of flow. The SV is seen joining the SMV to form the PV, which drains directly into the IVC. (D) Well-differentiated HCC arising in hepatocellular adenoma (H&E, 20×); the left inset demonstrates an area of well-differentiated HCC (H&E, 200×), and the right inset shows reticulin stain with loss of staining (H&E, 200×). (E) A small portal tract shows an arteriole and bile duct, but no portal vein, features that are consistent with Abernethy malformation (H&E, 100×). (F) Coronal contrast-enhanced CT image of the abdomen after orthotopic liver transplantation from a living-related donor demonstrates the main portal vein entering the liver at the hilum. Right intrahepatic portal veins are visualized. Note the right hepatic vein anastomosis to the inferior vena cava. Contrast this image to the pretransplant CT image in (B). CT, computed tomography; HCC, hepatocellular carcinoma; IVC, inferior vena cava; PV, portal vein; SMV, supermesenteric vein.

Contrast-enhanced computed tomography (CT) showed multiple hyperenhancing hepatic masses and a 5.5-cm right lobe lesion with enhancement, washout, and vascular anatomy compatible with CEPS (Figure 1). A 7-cm lesion suggested an FNH, and a 10.6-cm lesion had a hemorrhage suggestive of HCC (Figure 1). The unresectable disease burden and biopsy-proven HCC created the urgency for curative treatment with a living donor liver transplantation (LDLT).

Four months later, she underwent successful LDLT without any surgical complications. The liver explant showed 3 foci of well-differentiated HCC arising from HCAs without lymphatic space invasion, 2 HCAs, and 2 FNHs. The portal veins were absent in small portal tracts, hypoplastic portal veins in medium-sized and large-sized portal tracts, and isolated capillaries and arterioles in the lobules; findings that were compatible with CEPS (Figure 1).

One week after the transplant, serologies were remarkable for alkaline phosphatase 221 U/L, aspartate aminotransferase 490 U/L, alanine aminotransferase 445 U/L, and total bilirubin 1.6. All liver enzymes returned to normal, and her pruritus resolved within 2 months after the surgery.

Eighteen months after LDLT, she continues to take tacrolimus and low-dose prednisone, with excellent allograft function, no episodes of acute cellular rejection, HCC recurrence, or liver lesions on subsequent serial abdominal ultrasounds (Figure 1). She is also in her second trimester of pregnancy. She and the fetus are healthy.

## DISCUSSION

Another case report where HCA progressed to HCC in an adult female with CEPS involved a 20-year-old who had elevated sex hormones and primary amenorrhea.^5^ CT showed several large HCAs and a noncirrhotic liver with biopsy-proven HCC. Despite the recommendation to undergo a liver transplant, she chose transarterial chemoembolization and microwave ablation. Five months later, the lesions progressed, and she underwent bland embolization followed by microwave ablation, fortunately, this time with a good response.

Also rare is for FNH and HCC to coexist in an adult patient with CEPS. A 38-year-old woman with a body mass index (BMI) of 36 presented with right upper-quadrant abdominal pain and had elevated liver enzymes and normal liver tumor markers.^6^ She had enlarging nodules without cirrhosis on CT and underwent hepatic lobectomy. The biopsy showed FNH, fibrolamellar HCC, and Abernethy malformation Type I anatomy. Nine months later, she had increasing hepatomegaly and new nodules and underwent liver transplantation. The explant had no additional malignant nodules or FNH. At her 2-month follow-up, she was doing well without evidence of HCC recurrence.

Although usually limited to case reports, an international, multicenter observational study looked at 66 patients with CEPS, 12 of which had FNH and 8 had HCC.^7^ The median age was 39 years, and most HCC cases were identified 9 years after CEPS diagnosis. AFP was elevated in 5 of 29 patients, and 4 had HCC. Most had surgical hepatectomy, and 1 with hepatic encephalopathy underwent liver transplantation and was disease-free 6 months later.

Transformation of HCA to HCC occurs in less than 5% of cases.^8^ FNH is believed to have no malignant potential and rarely recurs after resection.^9^ Exposure to estrogenic or androgenic steroids is a risk factor for HCA and FNH, as seen in our patient and the case described above.^5,8^ The cases described here demonstrate that these lesions can exist simultaneously, and HCC can arise within an adenoma in adult patients with CEPS.

Proposed mechanisms by which HCA and FNH form in patients with CEPS include pancreatic hormones flowing through the portal bloodstream that induce hepatocyte regeneration, increased arterial flow leading to nodules, and absence of portal hepatic perfusion modifying hepatocytes.^5,10^ Immunophenotypic analyses have identified identical genes between HCA and HCC, evidence that erroneous genetic alterations in HCA could lead to HCC.^11^

Our case features the presentation and progression of tumors in adults with CEPS and highlights that HCA, FNH, and HCC can coincide despite AFP being normal. We make a case for serial diagnostic imaging to monitor enlarging or atypical hepatic lesions in patients with CEPS who have FNH and HCA. Suspicious lesions may be evaluated with biopsy, given that AFP is often normal in the presence of HCC. We demonstrate that liver transplantation should be considered for patients with high-risk liver lesions not amenable to surgical resection.

Although most CEPS cases are detected in childhood, complications can arise in adulthood, leading to a delayed diagnosis.^4^ Future research is warranted about the presentation, progression, and complications experienced by adults with CEPS. Investigations with large cohorts from multiple centers can help tailor the optimal surveillance and treatment plans for this select population.

## DISCLOSURES

Author contributions: CK Tom: conception and design, acquisition, analysis, interpretation, drafting the work, final approval of the version to be published, and agreement to be accountable for all aspects of the work. R. Ter-Oganesyan, C. Shefali, and N. Kaur: analysis, interpretation, reviewing the work critically, final approval of the version to be published, and agreement to be accountable for all aspects of the work. JL Pace, A. Rastegarpour, and Y. Genyk: reviewing the work critically, final approval of the version to be published, and agreement to be accountable for all aspects of the work. JA Kahn: conception and design, reviewing the work critically, final approval of the version to be published, agreement to be accountable for all aspects of the work, and is the article guarantor.

Financial disclosure: None to report.

Previous presentation: This case report has been presented at the ACG 2023 Annual Scientific Meeting for poster presentation; October 24, 2023; Vancouver, Canada.

Informed consent was obtained for this case report.

## References

[1] Alonso-Gamarra E Parrón M Pérez A Prieto C Hierro L López-Santamaría M. Clinical and radiologic manifestations of congenital extrahepatic portosystemic shunts: A comprehensive review. Radiographics. 2011;31(3):707–22. 21571652 10.1148/rg.313105070

[2] Bernard O Franchi-Abella S Branchereau S Pariente D Gauthier F Jacquemin E. Congenital portosystemic shunts in children: Recognition, evaluation, and management. Semin Liver Dis. 2012;32(4):273–87. 23397528 10.1055/s-0032-1329896

[3] Kim MJ Ko JS Seo JK Clinical features of congenital portosystemic shunt in children. Eur J Pediatr. 2012;171(2):395–400. 21912894 10.1007/s00431-011-1564-9

[4] Christou N Dib N Chuffart E Stepwise management of hepatocellular carcinoma associated with Abernethy syndrome. Clin Case Rep. 2018;6(5):930–4. 29744090 10.1002/ccr3.1384 PMC5930207

[5] Chiang J Chiu HK Moriarty JM McWilliams JP. Hyperandrogenism and malignant degeneration of hepatic adenomas in the setting of Abernethy malformation. Radiol Case Rep. 2020;15(12):2701–5. 33117471 10.1016/j.radcr.2020.10.026 PMC7581830

[6] Scheuermann U Foltys D Otto G. Focal nodular hyperplasia precedes hepatocellular carcinoma in an adult with congenital absence of the portal vein. Transpl Int. 2012;25(5):e67–e68. 22394294 10.1111/j.1432-2277.2012.01454.x

[7] Baiges A Turon F Simón-Talero M Congenital extrahepatic portosystemic shunts (Abernethy malformation): An international observational study. Hepatology. 2020;71(2):658–69. 31211875 10.1002/hep.30817

[8] Stoot JH Coelen RJ De Jong MC Dejong CH. Malignant transformation of hepatocellular adenomas into hepatocellular carcinomas: A systematic review including more than 1600 adenoma cases. HPB (Oxford). 2010;12(8):509–22. 20887318 10.1111/j.1477-2574.2010.00222.x PMC2997656

[9] Balabaud C Al-Rabih WR Chen PJ Focal nodular hyperplasia and hepatocellular adenoma around the world viewed through the scope of the immunopathological classification. Int J Hepatol. 2013;2013:268625. 23691331 10.1155/2013/268625 PMC3654480

[10] Starzl TE Francavilla A Halgrimson CG The origin, hormonal nature, and action of hepatotrophic substances in portal venous blood. Surg Gynecol Obstet. 1973;137(2):179–99. 4353133 PMC2747591

[11] Ercan C Coto-Llerena M Gallon J Genomic analysis of focal nodular hyperplasia with associated hepatocellular carcinoma unveils its malignant potential: A case report. Commun Med (Lond). 2022;2:11. 35603298 10.1038/s43856-022-00074-y PMC9053256

